# The Hygiene of Transmissable Diseases; Their Causation, Modes of Dissemination and Methods of Prevention

**Published:** 1901-09

**Authors:** 


THE HYGIENE OF TRANSMISSABLE DISEASES; THEIR CAUSATION, MODES OF DISSEMINATION AND METHODS OF PREVENTION. By A. C. Abbott, M. D., Professor of Hygiene and Bacteriology, University of Pennsylvania. Third edition, revised and enlarged. Octavo, 351 pages, with numerous illustations. Philadelphia and London: W. B. Saunders & Co. Cloth, $2.50 net.

This follows closely the plan of the former edition, but the recent additions to our knowledge in the important field covered by the work have made necessary the complete revision and enlargement of some of the parts, and numerous minor changes in others. For example, the sections on malaria, yellow fever, plague, dysentery and tuberculosis have been entirely rewritten and have had much new matter added.

Proper attention has been paid to the rôle played by insects and rodents as disseminators of disease, and the established facts in this connection have been given the prominence they deserve.

In the study of a given disease, yellow fever for instance, the author considers its definition, etiology, geographical distribution and prophylaxis. Under these sub-headings are contained all the facts likely to be of interest to the student of practical hygiene.

